# Management of amphotericin-induced phlebitis among HIV patients with cryptococcal meningitis in a resource-limited setting: a prospective cohort study

**DOI:** 10.1186/s12879-019-4209-7

**Published:** 2019-06-26

**Authors:** Cynthia Ahimbisibwe, Richard Kwizera, Jane Frances Ndyetukira, Florence Kugonza, Alisat Sadiq, Kathy Huppler Hullsiek, Darlisha A. Williams, Joshua Rhein, David R. Boulware, David B. Meya

**Affiliations:** 1 Infectious Diseases Institute, College of Health Sciences, Makerere University, Kampala, Uganda; 2 Division of Biostatistics, School of Public Health, University of Minnesota, Minneapolis, USA; 3 Division of Infectious Diseases and International Medicine, Department of Medicine, University of Minnesota, Minneapolis, MN USA; 4 Department of Medicine, School of Medicine, College of Health Sciences, Makerere University, Kampala, Uganda

**Keywords:** Phlebitis, Thrombophlebitis, Amphotericin B, Cryptococcal infection, HIV, Nursing, Sub-Saharan Africa

## Abstract

**Background:**

Amphotericin-induced phlebitis is a common infusion-related reaction in patients managed for cryptococcal meningitis. High-quality nursing care is critical component to successful cryptococcosis treatment. We highlight the magnitude and main approaches in the management of amphotericin-induced phlebitis and the challenges faced in resource-limited settings.

**Methods:**

We prospectively determined the incidence of amphotericin-induced phlebitis during clinical trials in Kampala, Uganda from 2013 to 2018. We relate practical strategies and challenges faced in clinical management of phlebitis.

**Results:**

Overall, 696 participants were diagnosed with HIV-related cryptococcal meningitis. Participants received 7–14 doses of intravenous (IV) amphotericin B deoxycholate 0.7–1.0 mg/kg/day for induction therapy through peripheral IV lines at a concentration of 0.1 mg/mL in 5% dextrose. Overall, 18% (125/696) developed amphotericin-induced phlebitis. We used four strategies to minimize/prevent the occurrence of phlebitis. First, after every dose of amphotericin, we gave one liter of intravenous normal saline. Second, we rotated IV catheters every three days. Third, we infused IV amphotericin over 4 h. Finally, early ambulation was encouraged to minimize phlebitis. To alleviate phlebitis symptoms, warm compresses were used. In severe cases, treatment included topical diclofenac gel and oral anti-inflammatory medicines. Antibiotics were used only when definite signs of infection developed. Patient/caregivers’ education was vital in implementing these management strategies. Major challenges included implementing these interventions in participants with altered mental status and limited access to topical and oral anti-inflammatory medicines in resource-limited settings.

**Conclusions:**

Amphotericin-induced phlebitis is common with amphotericin, yet phlebitis is a preventable complication even in resource-limited settings.

**Trial registration:**

The ASTRO-CM trial was registered prospectively. ClincalTrials.gov: NCT01802385; Registration date: March 1, 2013; Last verified: February 14, 2018.

## Background

Cryptococcal meningitis accounts for 15 to 25% of AIDS-related deaths [1–3]. Treatment of cryptococcal meningitis currently relies on amphotericin B based regimens. The 2018 World Health Organization (WHO) guidelines recommend a one-week induction regimen with amphotericin B deoxycholate and flucytosine for treating cryptococcal meningitis among human immunodeficiency virus (HIV) patients [4]. However, in the absence of flucytosine, 2 weeks of amphotericin B deoxycholate and high dose fluconazole is recommended. This is followed by a consolidation and maintenance phase using oral fluconazole monotherapy.

In high-resource settings, intravenous (IV) administration of amphotericin B deoxycholate is typically given through a central line. However, in resource-limited settings, amphotericin is typically administered via peripheral venous access due to limited supplies. Amphotericin has toxicity with severe side effects [5]. These infusion-related side effects include nephrotoxicity, nausea, vomiting, fevers, chills, hypotension and phlebitis [6]. Phlebitis is a common, potentially severe infusion-related reaction. Phlebitis may be directly associated with infusion of amphotericin, as well as other intravenous medications commonly used to prevent amphotericin-induced toxicity, such as potassium chloride. Phlebitis involves inflammation of the veins due to damage to the blood vessel walls, coagulation abnormality, or impaired venous blood flow. Phlebitis is characterized by pain, redness, tenderness and swelling of the affected area at the infusion site. In severe cases, infusion-associated phlebitis can lead to painful discomfort for the patient, undesirable interruption of the course of amphotericin treatment, possible thrombosis, or sepsis. All of these could negatively affect treatment outcomes.

High-quality nursing care is a critical component when administering amphotericin B for many AIDS-related opportunistic infections, including cryptococcosis, histoplasmosis, or talaromycosis [7]. Herein, we prospectively determined the incidence of amphotericin-induced phlebitis and described our practical experiences and challenges in managing it in a resource-limited setting during a prospective multi-site randomized clinical trial managing participants co-infected with HIV and cryptococcal meningitis.

## Methods

The current study reported in this manuscript was an observational cohort study that was nested under the Adjunctive Sertraline for the Treatment of HIV-associated Cryptococcal Meningitis (ASTRO-CM) Clinical Trial (ClincalTrials.gov: NCT01802385) [8]. Therefore, we adhered to the STROBE guidelines. Eligibility for this observational study was the same as that for the ASTRO-CM trial. We enrolled a prospective cohort of subjects screened for the ASTRO-CM Clinical Trial. This encompassed a phase II trial followed by a phase III randomized clinical trial conducted in Uganda at the Mulago National Referral and Mbarara Hospitals from Sept 2013 to May 2017 [8]. As part of this trial, all persons presenting with suspected meningitis were approached for consent for a diagnostic lumbar puncture. Participants included in the study were HIV-infected, ≥18 years, with a positive CSF CrAg.

Participants were diagnosed with cryptococcal meningitis based on cryptococcal antigen lateral flow antigen (Immy, Norman, Oklahoma) and confirmed with quantitative cerebrospinal fluid (CSF) culture [9, 10]. During the induction phase, patients with confirmed cryptococcal meningitis were treated primarily with amphotericin B deoxycholate 0.7–1.0 mg/kg/day via peripheral intravenous (IV) access at a concentration of 0.1 mg/mL in 5% dextrose. Amphotericin 50 mg was prepared in 500 mL. When participants weighed < 50 kg, volume was wasted before infusion to achieve 1.0 mg/kg dose. Participants received fluconazole 800-1200 mg per day and other concomitant medications aimed at mitigating the toxic effects of amphotericin (potassium chloride, magnesium chloride, multivitamins, analgesics, and ondansetron anti-emetic) per standardize treatment protocols.

Each participant received 7–14 doses of amphotericin. One liter of saline was administered intravenously immediately prior to amphotericin administration to minimize renal toxicity, and a second 1 liter was administered post-infusion to minimize phlebitis. During this cohort, KCl tablets (16 mEq) were given to all participants daily up to the end of the induction phase. However, for those that developed severe hypokalemia, we added IV KCl (40 mEq) in NaCl 0.9% (saline). Less than ten patients (out of 696) used the IV KCl. Clinicians and nurses reviewed participants daily to assess phlebitis, toxicity and patients’ clinical progress. Clinical reviews included taking vital signs, general examination, drug adherence, adverse events, reviewing/requesting laboratory tests, and recording any new complaints. In this study, we aimed to determine the incidence of amphotericin-induced phlebitis and describe our practical experiences and challenges in managing it in a resource-limited setting using these enrolled patients.

### Ethical consideration

All research participants or their surrogates provided written informed consent. Ethical approval was obtained from the Uganda National Council of Science and Technology (UNCST), Mulago Hospital Research and Ethics Committee, and the University of Minnesota.

### Statistical analysis

Data were analysed using STATA version 14 (STATA, College Station, Texas). We performed statistical analysis to determine the frequency of phlebitis and the distribution of baseline demographic characteristics of the participants. Summaries were made in frequency & percentages for each baseline characteristic considered as a categorical, and medians (interquartile range) when each characteristic is considered as a continuous variable.

## Results

### Summary of participants

During the ASTRO-CM study, 1739 participants were screened and 696 HIV-infected patients with cryptococcal meningitis were enrolled, of which 40% were women. The median age for all participants was 35 years (IQR; 30–41). The median CD4 cell count for all participants was 16 cells/μL (IQR; 6–49). The duration of hospitalization was a median of 14 days (IQR; 9–16). Over the duration of the study, 607 participants presented with an index episode of cryptococcal meningitis while 89 participants presented as second episodes (Table 1).

**Table 1:** Demographics of cryptococcal meningitis research participants

Demographics	Overall		Phlebitis During Induction		No Phlebitis During Induction		*P*-value
	N	Median [IQR] or N (%)	N	Median [IQR] or N (%)	N	Median [IQR] or N (%)	
Age, years	696	35 [30, 41]	125	35 [30, 40]	571	35 [30, 41]	0.53
Women	696	275 (39.5%)	125	47 (38%)	571	228 (40%)	0.63
Weight, kg	573	52 [48, 60]	97	53 [49, 60]	476	52 [48, 60]	0.82
Receiving HIV therapy	695	367 (53%)	125	59 (47%)	570	308 (54%)	0.17
Glasgow Coma Score < 15	695	295 (42%)	125	47 (38%)	570	248 (44%)	0.23
Duration of HIV, months	656	4.3 [0.3, 38.1]	116	4.1 [0.3, 50.8]	540	4.3 [0.3, 37.2]	0.79
CD4 T cell count, cells/μL	661	16 [6, 49]	122	15 [6, 50]	539	17 [6, 49]	0.63
Initial Meningitis episode	696	607 (87%)	125	118 (94%)	571	489 (86%)	**< 0.01**
Second Meningitis episode	696	89 (13%)	125	7 (5.6%)	571	82 (14%)	
CSF Culture, log_10_ CFU/mL	692	4.6 [2.8, 5.4]	124	4.5 [3.1, 5.3]	568	4.6 [2.8, 5.4]	0.89
Sterile cryptococcal culture	692	74 (11%)	124	5 (4.0%)	568	69 (12%)	**< 0.01**
CSF opening pressure, cm H_2_O	607	27 [18, 40]	105	27 [19, 38]	502	27 [18, 41]	0.63
Opening pressure > 25 cm H_2_O	607	336 (55%)	105	55 (52%)	502	281 (56.%)	0.50
CSF white cells/μL	671	< 5 [< 5, 45]	121	< 5 [< 5, 45]	550	< 5 [< 5, 45]	0.12
CSF white cells > 5 cells/μL	671	243 (36%)	121	36 (30%)	550	207 (38%)	0.10
CSF protein, mg/dL	594	49 [23, 100]	118	42 [25, 100]	476	50 [22, 100]	0.83

Data presented are N (%) or medians with interquartile ranges (IQR). Abbreviations: *CSF* cerebrospinal fluid, *CFU* colony forming units of *Cryptococcus*, *N* number of participants with data for each parameter

### Incidence of phlebitis

Overall, 18% (125/696) of participants developed amphotericin-induced phlebitis during hospitalization. Among the patients presenting with an index episode of cryptococcal meningitis, 19% (118/607) developed phlebitis while only 8% (7/89) of those with second episode meningitis developed phlebitis (*P* < 0.01). In our cohort, the time to development of phlebitis varied widely with 27% of the participants developing it in the first week of therapy while majority (43%) developed it in the second week of therapy. However, 30% of the participants developed phlebitis after the 2 weeks induction phase (Table 2).

**Table 2:** Duration of amphotericin B therapy and timing of Phlebitis during Cryptococcal meningitis

Characteristic	Phlebitis	No Phlebitis
N	125	571
Days of amphotericin B	14 [12, 14]	8 [4, 12]
Days in hospital	15 [14, 18]	14 [8, 16]
Time to phlebitis		
1–7 days	34 (27%)	
8–10 days	23 (18%)	
11–14 days	31 (25%)	
15–18 days	22 (18%)	
> 18 days	15 (12%)	
Received antibiotic for phlebitis^a^	8 (6.4%)	23 (4.0%)
Time to antibiotic initiation, days	13 [12, 15]	9 [6, 11]

^a^Limited to amoxicillin, cloxacillin, and doxycycline

Among demographic factors, the only difference was that baseline CSF culture sterility was associated with less phlebitis (P < 0.01) (Table 1). As part of standardized care, participants with sterile CSF culture only received 7 days of amphotericin thus were less at risk of phlebitis. Only 6% (8/125) of the participants with phlebitis and 4% (23/571) without phlebitis were given antibiotics during induction due to suspected presence of an infected thrombus. However, even with the increased awareness and attention we gave to phlebitis, its incidence did not reduce over time and the distribution by year was as follows; 21% in 2013 (26 cases), 13% in 2014 (16 cases), 8% in 2015 (10 cases), 26% in 2016 (33 cases) and 32% in 2017 (40 cases). This increased rate in 2016–2017 may reflect an observation bias of increased awareness and attention to phlebitis.

## Discussion

### Post amphotericin saline infusion

One liter or more of saline was administered intravenously immediately after amphotericin administration to minimize phlebitis and renal toxicity. Similarly, WHO recommends administration of “one liter of normal saline solution with 20 mEq of potassium chloride (KCl) over two hours before each controlled infusion of amphotericin B and one to two 8-mEq KCl tablets orally twice daily” [4]. The supplemental KCl is used to prevent hypokalemia and renal insufficiency resulting of potassium wasting. This strategy of giving saline also helped in managing dehydration in this population.

### Rotation of the IV infusion site

Each amphotericin infusion was regulated to run for at least 4 hours at a rate of approximately 120 mL/hr. (40 drops/ minute). However, in our setting, we do not use a standard infusion pump as would be in a higher-resourced environment. We use the regulator that is fixed on the IV tubing to help standardize the drip rate. The infusion should not be given too quickly given risk for both rigors and phlebitis with too rapid of an infusion. Previous studies have shown that Infusion concentrations of more than 0.1 g/L are associated with increased risks of amphotericin-induced phlebitis; which may be minimized by using a central line [11].

Time to development of amphotericin-induced phlebitis and the factors that affect this time remain unclear. This is because some patients developed the inflammation after one dose while others developed it after additional doses. Previous studies showed that IV antibiotics, female sex, prolonged (> 48 h) catheterization and catheter material strongly predicted phlebitis [12]. Another recent study indicated that the incidence of phlebitis was associated with the length of time the catheter remained in place and puncture in the forearm [13].

The IV infusion site was changed after every 3 days or when patients complained of severe pain at the site of IV access or limb swelling, which suggested phlebitis. In most cases, the IV infusion was moved to the other arm, which would give time for the swelling on the arm previously used to resolve. However, in a few cases, both arms would have prolonged phlebitis that required us to move the IV line to the lower limbs to prevent treatment interruption. The main challenge to this method was that patients were usually dehydrated, making it difficult to find a vein to place a cannula for IV infusion. There is also limited ability and capacity to perform venous cut-downs or place central venous catheters. So none of the patients got a central venous catheter. In addition, some patients were disoriented and aggressive. This made it difficult to obtain intravenous access or sustain access. Finally, shortage of infusion sets to allow intravenous access rotation every 3 days is a challenge outside of the research setting in many resource-limited settings.

### Ambulation

Following the amphotericin infusion, we encouraged the patients to move out of bed and engage in light activity such as walking, sitting or standing as soon as the IV infusions for the day were completed. We educated the patients’ caregivers to help us implement this practice since most of the patients were semi-conscious and not well oriented. However, this approach was not possible for patients who were unconscious or comatose secondary to cryptococcal meningitis. The most challenging patients were those who were unconscious and had no caregivers. In such cases, we engaged the hospital social workers and physiotherapists to assist with patient ambulation and general care. However, the social workers and physiotherapist are generally limited and not readily available. This method of ambulation also helped to avoid bedsores among the bed-ridden patients.

### Treatment of phlebitis

#### Use of a warm compress

In addition to ambulation, we educated the patient caregivers to use a warm compress. This is a traditional method that involves applying a wet and warm clean cloth to the inflamed sites while applying pressure (compresses). This method was also recently described to be effective and inexpensive in the management of phlebitis [14]. This was the easiest and most convenient method for the caregivers in our setting. We encouraged them to apply the warm compress at least twice daily. However, adherence to this method was limited by poor access to hot water during their inpatient stay. Patient attendants had to buy the hot water or occasionally had it delivered from home.

#### Topical anti-inflammatory drugs

Topical anti-inflammatory drugs were applied to manage phlebitis in some patients who did not respond well to ambulation or warm compresses. We mainly used topical diclofenac gel and Emami mentho plus® balm, which provided quick relief compared to oral analgesics. Almost all the patients used them. However, these drugs were not freely available and were unaffordable for some patients or caregivers.

#### Oral anti-inflammatory medication

Oral anti-inflammatory drugs are recommended for symptomatic management of amphotericin infusion reactions including phlebitis [6]. In our setting, we routinely administered paracetamol (acetaminophen) in this population to manage headache, chills, fevers, rigors, and phlebitis, all of which are caused by amphotericin infusions. Non-steroidalanti-inflammatory drugs were not used due to the increased risk of renal toxicity with amphotericin co-administration. However, the effectiveness of paracetamol in the management of phlebitis remains unclear and warrants further investigation. The oral route of administration was also a challenge in unconscious patients. In such cases, we inserted a nasogastric tube for drug administration and feeding.

#### Oral antibiotics

There were patients who developed grade 4 phlebitis (“pain at the puncture site with erythema, hardening and a palpable venous cord that is >1 cm, with purulent discharge” [13]) that were persistent, locally advanced, associated with systemic symptoms such as fever, and did not respond to any of the management methods described above. In such cases, we assumed the presence of an infected thrombus and provided antibiotics for these patients. We primarily used oral antibiotics with Gram-positive coverage such as ampicillin-cloxacillin, amoxicillin-clavulanate, or doxycycline to cover possible staphylococcal infection for 5 days. Only 6% of the participants with phlebitis and 4% without phlebitis were given antibiotics during hospitalization. We generally avoided antibiotics unless definite signs of infection were present in accordance with good practice and standards of antimicrobial stewardship.

### Limitations to the study

The data and perspective presented herein are based on our experiences in Kampala, Uganda during a longitudinal, multisite cryptococcal meningitis trial. The research support enabled more resources to be available than in routine care. Additional management strategies may be available. Phlebitis and bacterial infections may be a contribution as to why 1 week of amphotericin and flucytosine has lower mortality than 2 weeks of amphotericin [15]. The investigation of single dose 10 mg/kg of liposomal amphotericin B may also have a distinct benefit of avoiding phlebitis [16, 17].

## Conclusion

We have demonstrated that in this Ugandan cohort of HIV-infected patients with cryptococcal meningitis who were treated with amphotericin B deoxycholate, phlebitis occurred in 18% of patients. However, the management of phlebitis is very challenging in resource-limited settings. We recommend slow intravenous infusion preferably using standard infusion pumps, with routine post amphotericin saline infusion and rotating the IV access site every 3 days to minimize phlebitis. This should be supplemented by ambulation and use of warm compresses. In severe cases, topical and oral anti-inflammatory drugs may be used. Antibiotics are generally indicated only when signs of infection are observed. Patients’/caregiver education is very vital in implementing the management strategies. However, altered mentation, limited availability of essential drugs, and medical supplies remain a significant challenge for high-quality nursing care to implement these management strategies to manage amphotericin-induced phlebitis in routine care in resource-limited settings.

## Data Availability

The datasets used and/or analysed during the current study are available from the corresponding author on reasonable request.

## Abbreviations

AIDS: Acquired immune deficiency syndrome
ASTRO-CM: Adjunctive Sertraline for the Treatment of HIV-associated Cryptococcal Meningitis
CSF: Cerebrospinal fluid
HIV: Human immunodeficiency virus
IV: Intravenous
KCl: Potassium chloride
UNCST: Uganda National Council of Science and Technology
WHO: World Health Organization
